# Associations between body circumference and testosterone levels and risk of metabolic dysfunction-associated fatty liver disease: a mendelian randomization study

**DOI:** 10.1186/s12889-023-15467-4

**Published:** 2023-03-30

**Authors:** Lin Ning, Jianguang Sun

**Affiliations:** Department of Traditional Chinese medicine, The first clinical medical college, Shandong University of Traditional Chinese Medicine, Jinan, China

**Keywords:** Waist circumference_1_, Hip circumference_2_, Testosterone_3_, Metabolic dysfunction-associated fatty liver disease_4_, Two-sample mendelian randomization analysis_5_, Causal Inference_6_

## Abstract

**Backgroud:**

Body circumference and testosterone levels have been reported as associated with metabolic dysfunction-associated fatty liver disease (MAFLD) risk. However, whether body circumference and testosterone levels play a role in the development of MAFLD remains inconclusive.

**Methods:**

Using a large database of genome-wide association studies, genetic loci that are independent of each other and strongly associated with body circumference and testosterone levels were selected as instrumental variables, the causal relationship between body circumference and testosterone and risk of MAFLD was investigated by two-sample Mendelian randomization methods such as inverse variance weighted (IVW), MR-Egger regression, and weighted median estimator (WME), using the odds ratios (ORs) as evaluation indicators.

**Results:**

A total of 344 SNPs were included as instrumental variables in this study, including 180 for waist circumference, 29 for waist-to-hip ratio, and 135 for testosterone levels. Using the above two-sample Mendelian Randomization method to derive the causal association between exposure and outcome. The results of this study showed that three exposure factors were causally associated with the risk of MAFLD. Waist circumference obtained three statistically significant results for IVW, WME and Weighted mode (IVW: OR = 3.53, 95%CI: 2.23–5.57, *P* < 0.001; WME: OR = 3.88, 95%CI: 1.81–8.29, *P* < 0.001; Weighted mode: OR = 3.58, 95%CI: 1.05–12.16, *P* = 0.043). Waist-to-hip ratio obtained one statistically significant result for IVW (OR = 2.29, 95%CI: 1.12–4.66, *P* = 0.022). Testosterone levels obtained one statistically significant result for IVW (OR = 1.93, 95%CI: 1.30–2.87, P = 0.001). Waist circumference, waist-to-hip ratio and testosterone level were considered as risk factors for MAFLD. The Cochran Q test for IVW and MR-Egger method indicated that there was no intergenic heterogeneity in SNPs. The test for pleiotropy indicated that the possibility of pleiotropy in the causal analysis was weak.

**Conclusion:**

The results of the two-sample Mendelian randomization analysis showed that waist circumference was the exact risk factor for MAFLD, waist-to-hip ratio and testosterone levels were potential risk factors for MAFLD, the risk of developing MAFLD increases with these three exposure factors.

**Supplementary Information:**

The online version contains supplementary material available at 10.1186/s12889-023-15467-4.

## Introduction

Metabolic dysfunction-associated fatty liver disease (MAFLD) was renamed from Non-alcoholic fatty liver disease (NAFLD) in March 2020 [1]. The disease is characterized by intrahepatocellular lipid deposition combined with systemic multisystemic metabolic disorders [2], the prevalence is increasing year by year and has become the number one chronic liver disease in China and the global prevalence is about 25% [3]. MAFLD can not only cause steatohepatitis and liver fibrosis, liver cancer, but also cause extra-hepatic complications such as cardiovascular disease and chronic kidney disease due to MAFLD-related metabolic disorders, which are more serious to people’s health [4]. However, so far, there are no effective preventive and therapeutic drugs for MAFLD, therefore, it is significant to identify the factors affecting the development of MAFLD and to intervene early for the development of MAFLD. Obesity, especially abdominal obesity, is the primary risk factor for MAFLD. Body circumference, especially waist circumference and waist-to-hip ratio, are practical indicators of body BMI and obesity[5]. Previous studies[6–8] have found that the incidence of fatty liver disease increases with increasing waist circumference and that waist-to-hip ratio is also a risk factor for the development of MAFLD [9]. It has also been shown that sex hormone levels are significantly associated with various specific disorders in the metabolic syndrome, testosterone is an important regulator of glucose and lipid metabolism in the body and is an intrinsic indicator of hepatic lipid metabolism [10–12], and the severity of disease in MAFLD patients is significantly correlated with blood testosterone levels [13]. However, the perspective of the above studies is limited to traditional observational epidemiology, which is vulnerable to unknown confounding factors and reverse causation. In recent years, mendelian randomization (MR) has been developed as an important method for causal inference, and it uses exposure-related genotypes as instrumental variables (IVs) and can overcome the drawbacks of traditional epidemiological studies such as difficult data acquisition and poor extrapolation of results [14]. However, traditional MR requires genotypes from the same individual, as well as information on exposure and outcome, and data detection costs are high. Recently, the two-sample Mendelian randomization (2-sample MR) method has been gradually developed, which allows gene and exposure association data and gene and outcome association data from two independent sample populations of the same overall population, respectively, compared with traditional MR, greatly improving the efficiency and feasibility of etiological studies, and has been widely used in causal association studies of risk factors and disease outcomes[15].

This study used a two-sample MR method to explore the causal associations between three exposure factors of body circumference (including waist circumference and waist-to-hip ratio) and testosterone level and risk of MAFLD.

## Materials and methods

### Study design

**Fig. 1:**
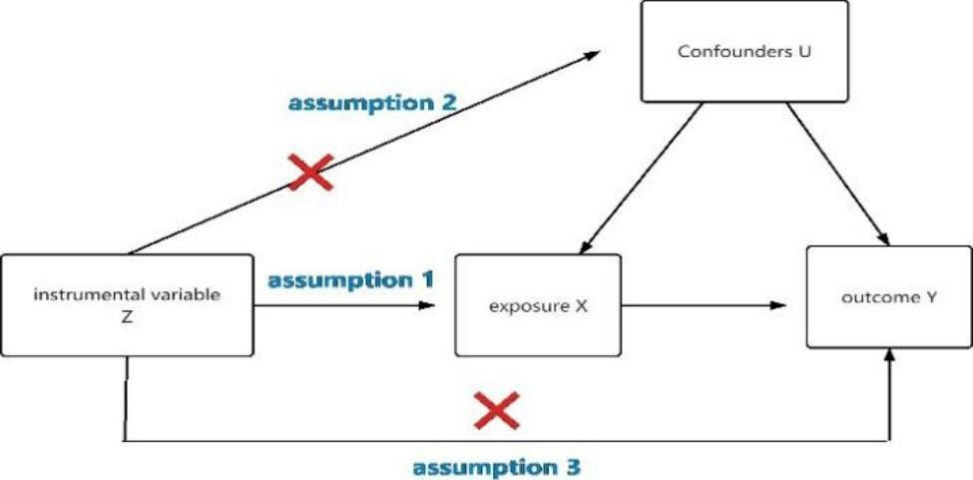
Model of the two-samples MR analysis

In this study, body circumference (waist circumference, waist-to-hip ratio) and testosterone levels were used as exposure factors, and single nucleotide polymorphisms (SNPs) loci significantly associated with the above exposure factors were selected as instrumental variables(IVs), and the outcome variable was MAFLD. The causal association analysis between exposure and outcome was performed using a two-sample MR analysis approach based on a publicly available genome wide association study (GWAS) database of large samples, and Cochran Q test to assess heterogeneity, and finally sensitivity analysis to verify the reliability of the causal association results. MR analysis needs to satisfy the following three core hypotheses: ①there is a strong association between instrumental variable Z and exposure factor X; ②instrumental variable Z is not associated with any confounding factor U of the exposure-outcome association; and ③the instrumental variable Z does not affect the outcome Y, except possibly by association with the exposure X. The two-sample MR study model is shown in Fig. 1.

### Data sources

In this study, significant body circumference (waist circumference, waist-hip ratio) and serum testosterone levels were used as exposure factors, SNPs significantly associated with the above exposure factors were used as IVs, and the outcome factor was MAFLD. The pooled data used to conduct the two-sample MR study were obtained from the IEU Open GWAS database summary website (https://gwas.mrcieu.ac.uk/ ), waist circumference (GWAS ID: ukb-a-382), waist-to-hip ratio (GWAS ID: ieu-a-79), testosterone (GWAS ID: ebi-a-GCST90012102), and MAFLD (GWAS ID: finn-b-NAFLD), all of the above databases were derived from European populations. All datasets used in this study were from the public domain, and summary information is presented in Table 1.

**Table 1:** Summary of the GWAS included in this two-sample MR study

Variable	ID	Sample size	Number of SNPs	Consortium	Population	Sex	Year
Waist circumference	ukb-a-382	336,639	10,894,596	Neale Lab	European	Males and Females	2017
Waist-to-hip ratio	ieu-a-79	210,082	2,542,432	GIANT	European	Males and Females	2015
Testosterone levels	ebi-a-GCST90012102	188,507	16,139,906	-	European	Males and Females	2020
MAFLD	finn-b-NAFLD	218,792	16,380,466	-	European	Males and Females	2021

### Selection of instrumental variables

SNPs with significant correlation with body circumference and testosterone level (*P* < 5. 0 × 10^− 8^ ) were screened, and the interference of linkage disequilibrium (LD) was excluded [16], setting parameter r^2^ = 0. 001, kb = 10,000, the echo SNPs and outlier SNPs were excluded, and the SNPs with significant heterogeneity were excluded by heterogeneity test. If the number of SNPs filtered according to the above criteria is large, each SNP should be queried on the PhenoScanner website (http://www.phenoscanner.medschl.cam.ac.uk/), SNPs affected by confounding factors that violated MR core hypothesis②and③were excluded. Finally valid SNPs significantly associated with exposure factors that met MR core hypothesis were obtained as IVs. *F* > 10 indicates the absence of weak instrumental variable bias, which is calculated as follows: $F = \frac{{N - k - 1}}{k} \times \frac{{{R^2}}}{{1 - {R^2}}}$, where N is the sample size of the exposed database, k is the number of SNPs, and R^2^ is the proportion of variance explained by SNPs in the exposed database. R^2^ is calculated as ${R^2} = \frac{{2 \times EAF \times \left( {1 - EAF} \right) \times {\beta ^2}}}{{S{D^2}}}$, where EAF is the effect allele frequency, β is the allele effect value, and SD is the standard deviation.

### Statistical analysis for mendelian randomization

We used the TwoSampleMR package (version 0.5.6) in R program (version 4.2.1) to integrate and analyze the data. In this study, inverse variance weighted (IVW) [17] was used as the main analysis method, while MR-Egger regression [18], weighted median estimator (WME) [19], simple mode and weighted mode [20] were used together for MR analysis. The principle of IVW is to weight the inverse of the variance of each IV as the weight while ensuring that all IVs are valid, the regression does not consider the intercept term, and the final result is the weighted average of the effect values of all IVs. The major difference between MR-Egger regression and IVW is that the regression takes into account the presence of the intercept term, and in addition, it also uses the inverse of the ending variance as a weight for the fit. The WME is defined as the median of the weighted empirical density function of the ratio estimates, which allows consistent estimation of causality if at least half of the valid instruments are present in the analysis.

### Heterogeneity and sensitivity test

There may be heterogeneity in the 2-sample MR analysis due to differences in analysis platforms, experimental conditions, including populations and SNPs, which may bias the estimation of causal effects. Therefore, the main IVW and MR-Egger methods were tested for heterogeneity in this study. The heterogeneity test was used to test the differences between individual IVs, and Cochran’s Q statistic and *P*-value were used to determine whether there was heterogeneity, and *P* < 0.1 represented the presence of heterogeneity; Pleiotropy test mainly tests the presence of horizontal pleiotropy for multiple IVs [21], and the *P*-value of the pleiotropy test was used in this study to measure whether there was pleiotropy in the analysis, if *P* > 0.05, it is considered that the possibility of pleiotropy in the causal analysis is weak. Leave-one-out sensitivity test is mainly to calculate the MR results of the remaining IVs after eliminating them one by one [22], if the estimated MR results of other IVs after eliminating one IV are very different from the total results, it means that the MR results are sensitive to that IV. The presence of pleiotropy in the analysis was also determined in this study using the MR-pleiotropy residual sum outlier (MR-PRESSO) [23].

## Results

### Instrumental variables

After screening SNPs with strong correlation with exposure in the corresponding GWAS database and removing the interference of linkage disequilibrium, we initially screened 418 SNPs, including 232 for waist circumference, 38 for waist-hip ratio, and 148 for testosterone level. Extracting the information of the above SNPs from the GWAS database corresponding to MAFLD, we obtained 412 valid SNPs, including 230 for waist circumference, 38 for waist-hip ratio, and 144 for testosterone level. Next, we eliminated echo SNPs and outlier SNPs, and finally we queried each SNP on the PhenoScanner website (http://www.phenoscanner.medschl.cam.ac.uk/) to exclude SNPs affected by confounding factors such as “alcohol consumption, body mass index, type 2 diabetes mellitus, hyperlipidemia, hypothyroidism”, etc. We eventually obtained 344 IVs, including 180 for waist circumference, 29 for waist-hip ratio, and 135 for testosterone level. The *F*-statistics corresponding to the single SNPs were all more than 10, indicating that the causal associations were less likely to be affected by weak instrumental variable bias. Basic information on the instrumental variables is in the *Supplementary Materials (Basic information on instrumental variables)*.

### Results of two-sample MR analysis

In this study, the IVW method was used as the main analytical method, and the other four MR analytical methods were complementary to the IVW method. In the MR analysis results, with an OR value bigger than 1, exposure was considered a risk factor for the outcome, and vice versa as a protective factor for the outcome, and when the *P* value was less than 0.05, it was considered statistically significant and the causal association was established. The results of the analysis in this study showed that all three exposure factors were causally associated with MAFLD. Waist circumference obtained three statistically significant results for IVW, WME and Weighted mode (IVW: OR = 3.53, 95%CI: 2.23–5.57, *P* < 0.001; WME: OR = 3.88, 95%CI: 1.81–8.29, *P* < 0.001; Weighted mode: OR = 3.58, 95%CI: 1.05–12.16, *P* = 0.043). Waist-to-hip ratio obtained one statistically significant result for IVW (OR = 2.29, 95%CI: 1.12–4.66, *P* = 0.022). Testosterone level obtained one statistically significant result for IVW (OR = 1.93, 95%CI: 1.30–2.87, *P* = 0.001). The above results suggest that waist circumference is a definite risk factor for MAFLD, and waist-to-hip ratio and testosterone levels are also potential risk factors for MAFLD, and as they increase, the risk of developing MAFLD also increases. The results of the specific analysis of the five methods are shown in Table 2. Visualization of MR results is shown in the *Supplementary Materials (MR results visualization charts)*.

**Table 2:** MR estimates of associations between 3 types of exposure and MAFLD risk

Exposure	Number of SNPs	MR methods	outcome	OR(95%CI)	*p-*value
Waist circumference	180	MR Egger	MAFLD	1.74 (0.42,7.22)	0.447
		WME		3.88 (1.81,8.29)	< 0.001
		IVW		3.53 (2.23,5.57)	< 0.001
		Simple mode		3.42 (0.57,20.42)	0.179
		Weighted mode		3.58 (1.05,12.16)	0.043
Waist-to-hip ratio	29	MR Egger		1.38 (0.04,44.83)	0.858
		WME		1.87 (0.69,5.12)	0.22
		IVW		2.29 (1.12,4.66)	0.022
		Simple mode		2.56 (0.39,16.62)	0.333
		Weighted mode		2.39 (0.495,11.49)	0.288
Testosterone level	135	MR Egger		1.67 (0.77,3.62)	0.199
		WME		1.71 (0.86,3.41)	0.125
		IVW		1.93 (1.30,2.87)	0.001
		Simple mode		2.32 (0.59,9.09)	0.227
		Weighted mode		1.58 (0.80,3.14)	0.192

Abbreviations: CI, confidence interval.

### Result of heterogeneity and sensitivity test

The Cochran Q test for IVW and MR-Egger method indicated that there was no intergenic heterogeneity in SNPs (*P* > 0.1); the test for pleiotropy indicated that the possibility of pleiotropy in the causal analysis was weak (*P* > 0.05), and the above results are detailed in Table 3.

**Table 3:** Results of Heterogeneity and sensitivity test

Exposure	outcome	MR methods	*p* of pleiotropy	*p* of Cochran Q
Waist circumference	MAFLD		0.305	
		MR Egger		0.204
		IVW		0.203
Waist-to-hip ratio			0.772	
		MR Egger		0.834
		IVW		0.864
Testosterone level			0.662	
		MR Egger		0.161
		IVW		0.173

In the visualization of MR results, the funnel plot showed that the points representing the causal association effect were roughly symmetrically distributed when a single SNP was used as the IV, indicating that the causal association was less likely to be affected by potential bias. The results of the “Leave-one-out” sensitivity analysis showed that after eliminating each SNP in turn, no SNP with a large effect on the causal association estimates was found.

## Discussion

In this study, the causal relationship between body circumference and testosterone level and risk of MAFLD was investigated using a two-sample MR analysis method using publicly available databases and a large-scale GWAS study. The results showed that all the exposure factors we studied were causally associated with the outcome, with waist circumference being the exact risk factor for MAFLD, waist-to-hip ratio and testosterone levels being potential risk factors for MAFLD, the risk of developing MAFLD increases with these three exposure factors. Our finding is consistent with the results of several previous studies. A five-year study by S Wang et al. including 12,477 observers showed that the cumulative incidence of MAFLD increased with increasing waist circumference and concluded that waist circumference was an independent risk factor for MAFLD [24]. A prospective cohort study in Korea involving 5400 people aged 40 ~ 69 years showed that waist circumference was the most important risk factor for MAFLD among the physical indicators of middle-aged and elderly people in Korea, and the threshold of waist circumference values were 81 cm for men and 78.5 cm for women [25]. Results of a clinical study on risk factors for NAFLD in Urumqi, China, showed that waist-to-hip ratio was a clinically meaningful risk factor for the development of NAFLD[26]. A clinical study of MAFLD in Taiwan, China, which included 1,969 participants, showed that waist-to-hip ratio was of concern in the incidence and severity of MAFLD, and that the correlation between waist-to-hip ratio and MAFLD was more pronounced in women than in men [27]. A Korean cohort study involving 613 women of various ages showed that serum testosterone levels in premenopausal women were positively associated with the risk of developing MAFLD [28]. The results of the above studies suggest that waist circumference, waist-to-hip ratio and testosterone levels are risk factors for MAFLD, which is consistent with the results of our study.

However, it should be noted that there are clinical studies showing gender differences in testosterone levels and the risk of MAFLD. A meta-analysis of 16 indicators involving 13,721 men and 5,840 women by Veeravich Jaruvongvanich et al. showed that the lower the testosterone level, the higher the risk of MAFLD in men, and in women, conversely, the lower the testosterone level, the lower the risk of developing MAFLD [29]. The multicenter clinical study by Monika Sarkar et al. showed that men with lower testosterone levels were more likely to develop MAFLD [30]. The above results also suggest that there may be gender differences in the causal association between testosterone levels and MAFLD, and the database of testosterone levels selected for this study includes both men and women, so the results of the analysis may be biased. To make the study results more reliable, the analysis should be performed separately according to gender.

This study explores the causal relationship between body circumference, testosterone, and risk of MAFLD using the two-sample MR method, which avoids the disturbance of confounding factors such as social environment and lifestyle because genetic variation is a long-term and stable exposure and can be measured directly. Compared to randomized controlled trials, MR allows for truly random assignment and does not violate ethics. Two-sample MR has a relatively larger sample size, allows for greater confidence, and increases the specificity of genetic variants using multiple IVs compared to a single SNP. The datasets used in this study were of large sample size and publicly available, which ensured the quality of the IVs used in the analysis. The IVs in this study fully considered and excluded SNPs that could affect the results, and outlier SNPs were excluded using the MR-PRESSO method. Therefore, the selection of IVs in this study is more detailed, comprehensive and reliable than previous studies. Of course, there are limitations in this study, as we did not perform a subgroup analysis by sex, and a more specific effect relationship could have been obtained with a two-sample MR analysis by sex, and this study has limitations in explaining the biological mechanisms underlying the causal effects of exposure and outcome.

In conclusion, this study validated the feasibility of applying MR methods to the study of the risk of developing MAFLD, and searching for key risk factors for preventable MAFLD at the genetic level, which has guiding implications for the early prevention of MAFLD in China. However, there are few GWAS data in Asian and Chinese populations, and data from other databases are difficult to obtain and organize, so the results of this study need to be further validated in Chinese populations in combination with clinical and randomized controlled trials.

## Electronic supplementary material

Below is the link to the electronic supplementary material.

MR results visualization charts

Basic information of instrumental variables

## Data Availability

The datasets [Exposure/Outcome] for this study can be found in the [IEU Open GWAS] [https://gwas.mrcieu.ac.uk/]
