# Supplementary material for: Associations between body circumference and testosterone levels and risk of metabolic dysfunction-associated fatty liver disease: a mendelian randomization study
Source: BMC Public Health. 2023 Mar 30;23:602. doi: 10.1186/s12889-023-15467-4 (PMC10061974; doi:10.1186/s12889-023-15467-4)

MR results visualization charts

Scatterplot of waist circumference and MAFLD risk

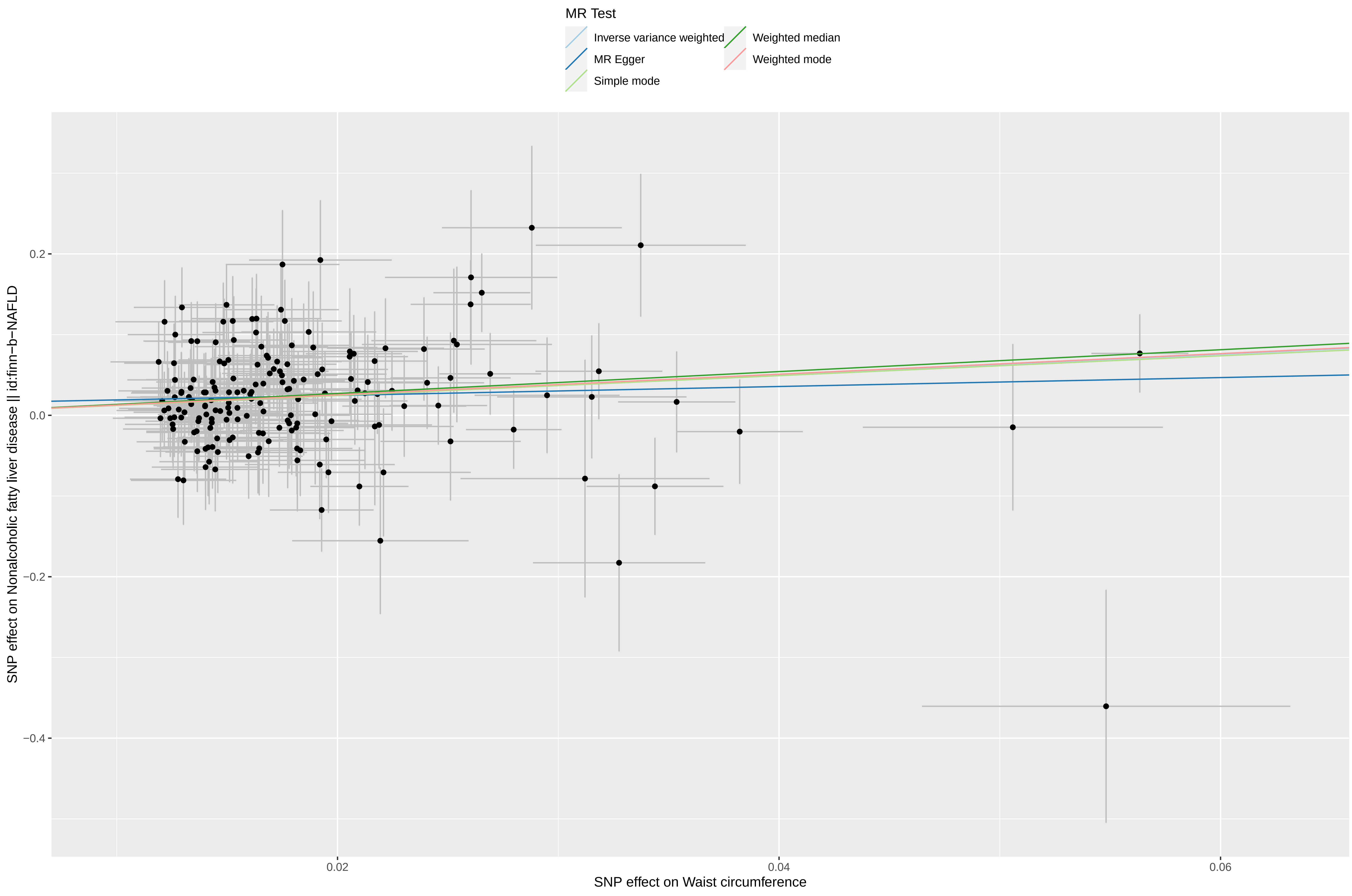

Funnel plot of waist circumference and MAFLD risk

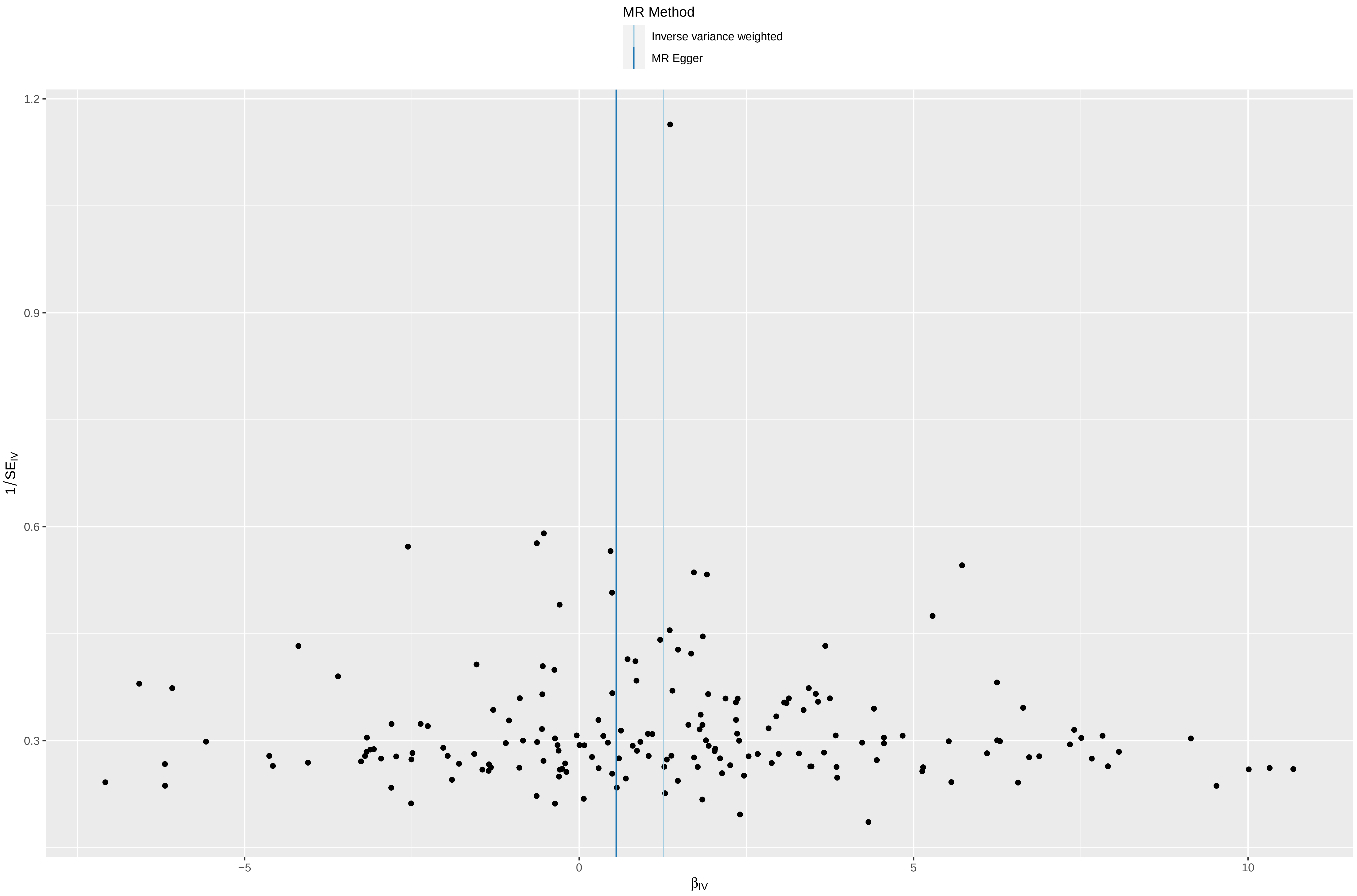

Forest plot of waist circumference and MAFLD risk

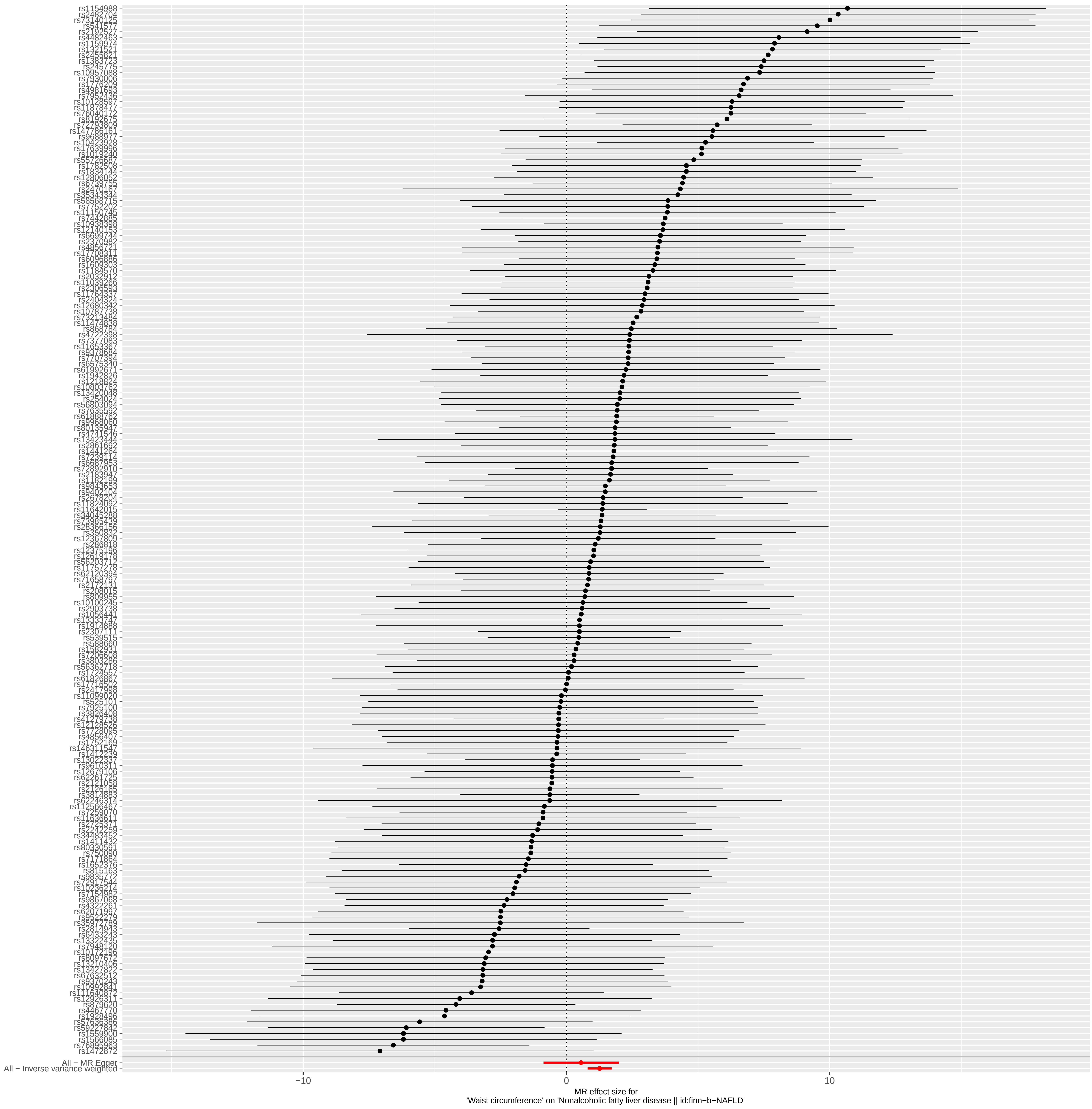

Sensitivity analysis of the "leave-one-out" method for waist circumference and MAFLD risk

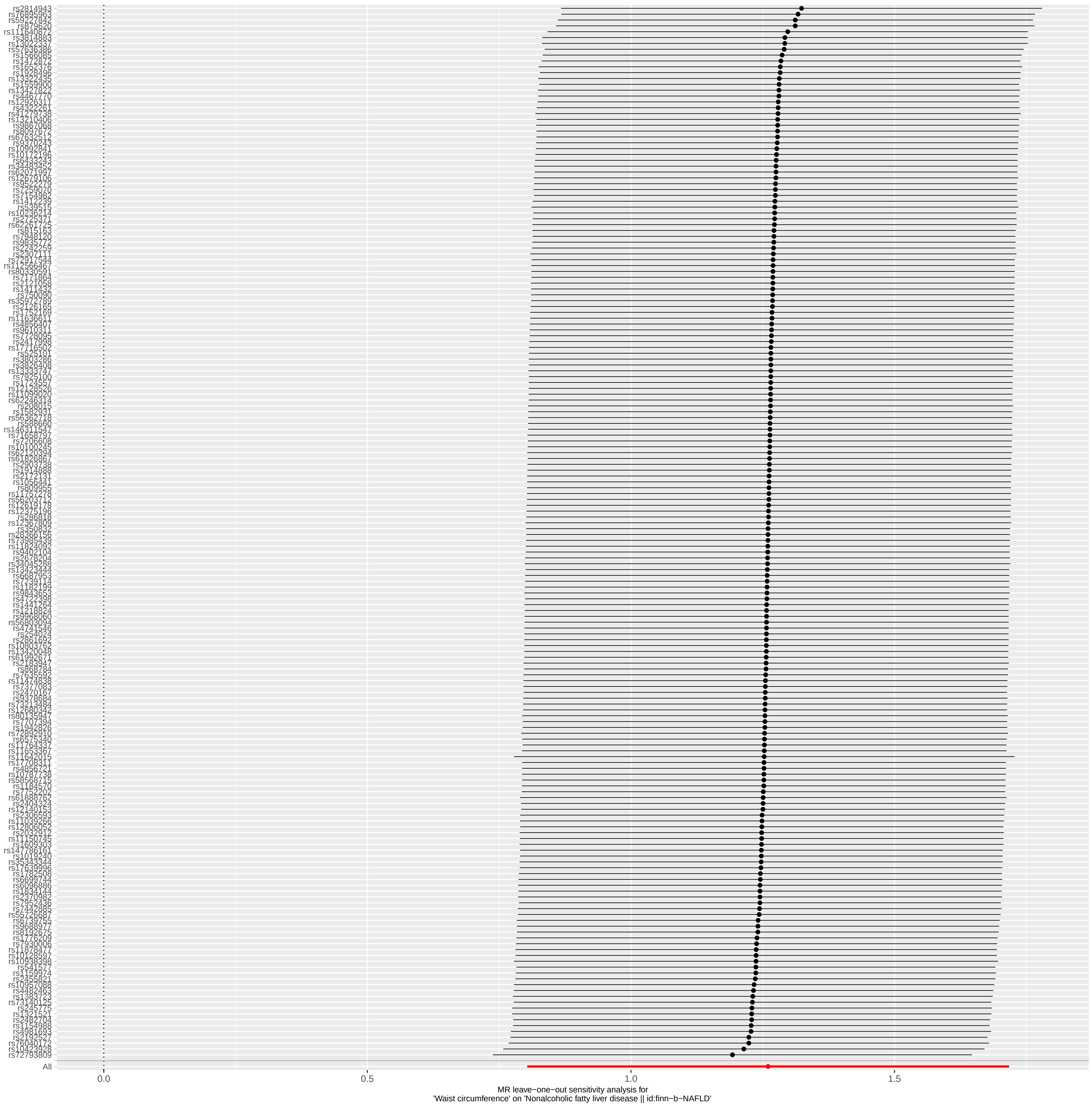

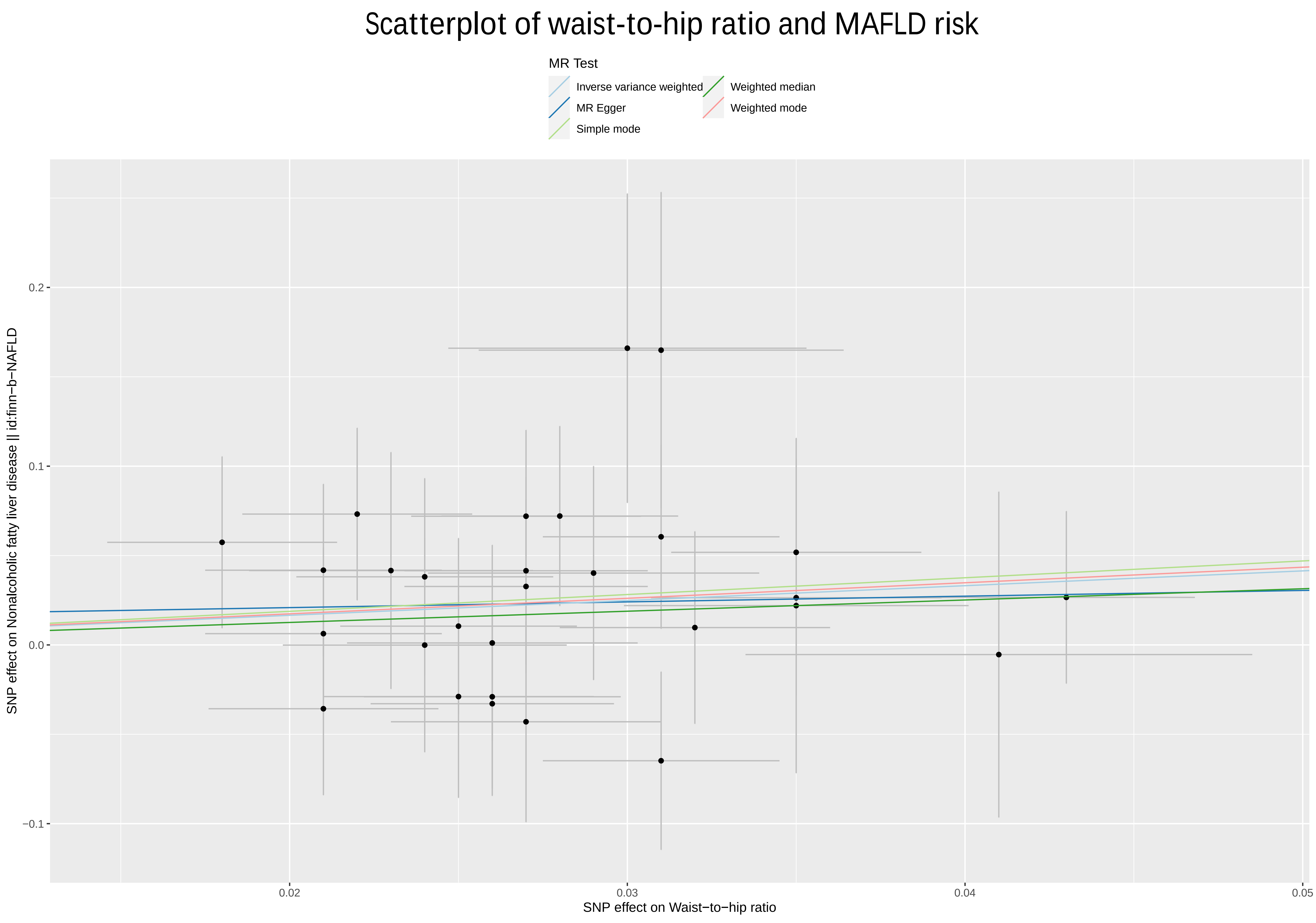
Scatterplot of waist-to-hip ratio and MAFLD risk
MR Test
Inverse variance weighted
MR Egger
Simple mode
Weighted median
Weighted mode
SNP effect on Nonalcoholic fatty liver disease || id:finn−b−NAFLD
0.2
0.1
0.0
−0.1
0.02
0.03
0.04
0.05
SNP effect on Waist−to−hip ratio

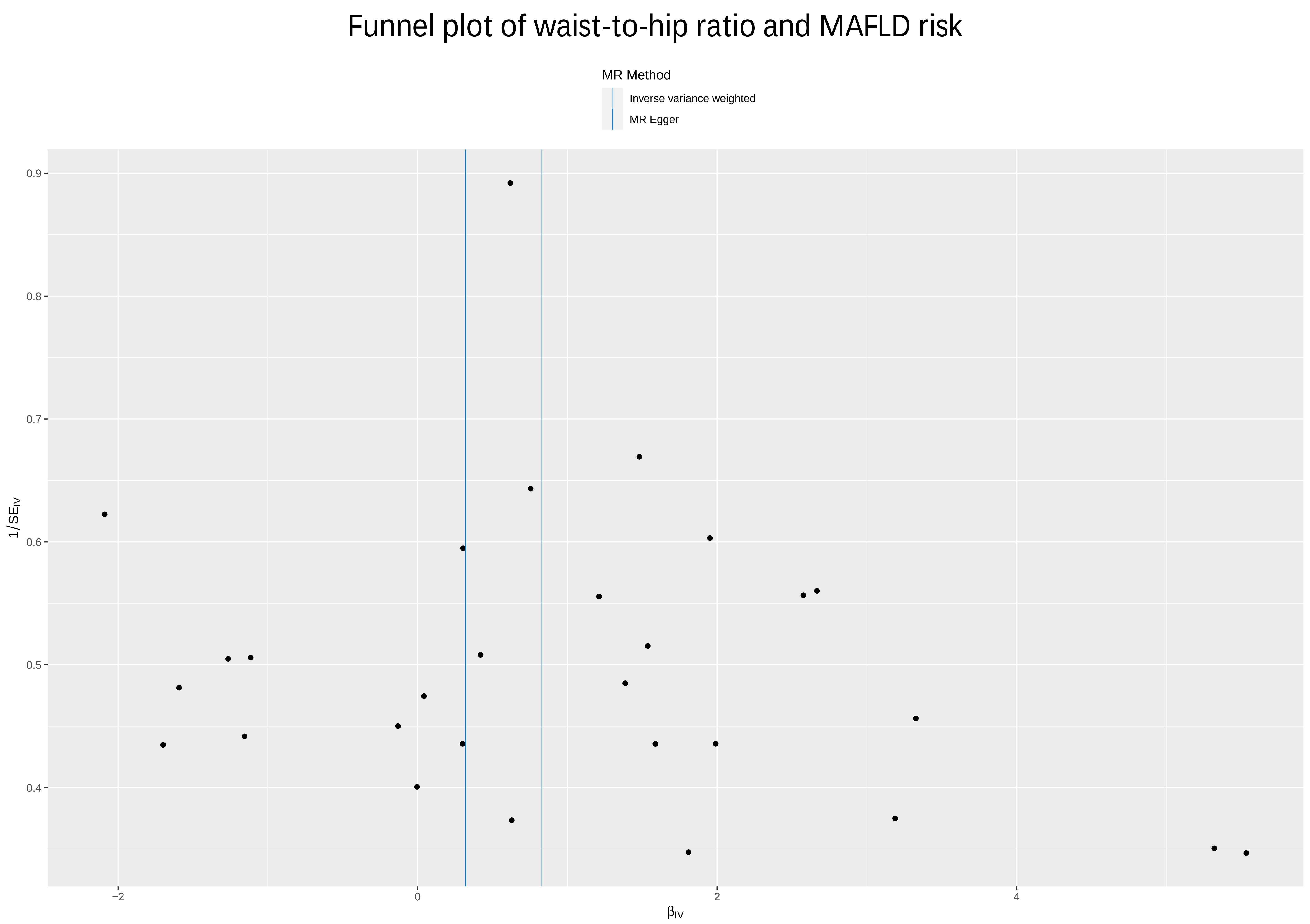
Funnel plot of waist-to-hip ratio and MAFLD risk
MR Method
Inverse variance weighted
MR Egger
1/SE_IV
0.9
0.8
0.7
0.6
0.5
0.4
−2
0
2
4
β_IV

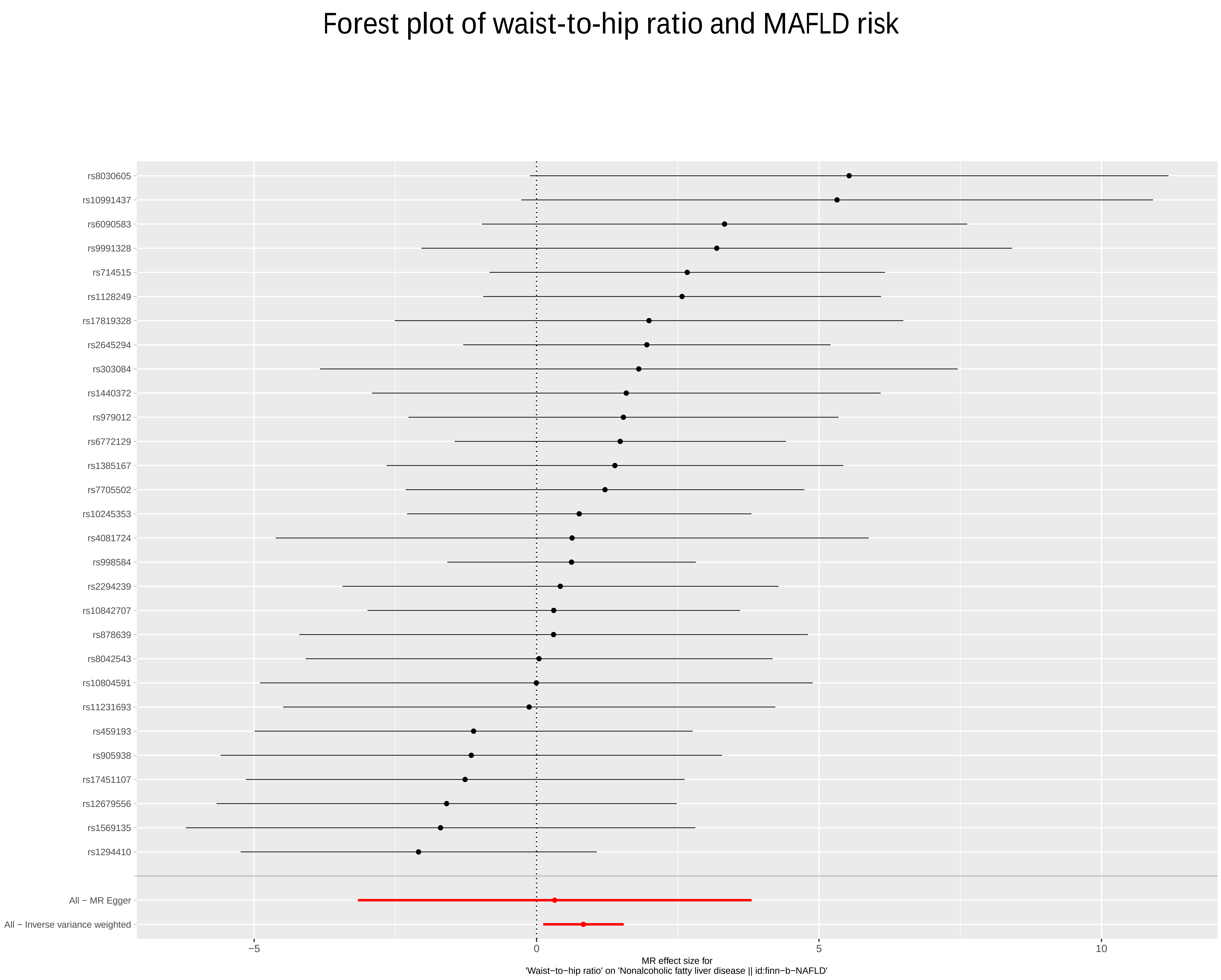

Forest plot of waist-to-hip ratio and MAFLD risk
rs8030605
rs10991437
rs6090583
rs9991328
rs714515
rs1128249
rs17819328
rs2645294
rs303084
rs1440372
rs979012
rs6772129
rs1385167
rs7705502
rs10245353
rs4081724
rs998584
rs2294239
rs10842707
rs878639
rs8042543
rs10804591
rs11231693
rs459193
rs905938
rs17451107
rs12679556
rs1569135
rs1294410
All − MR Egger
All − Inverse variance weighted
−5
0
5
10
MR effect size for
'Waist−to−hip ratio' on 'Nonalcoholic fatty liver disease || id:finn−b−NAFLD'

Sensitivity analysis of the "leave-one-out" method for waist-to-hip ratio and MAFLD risk

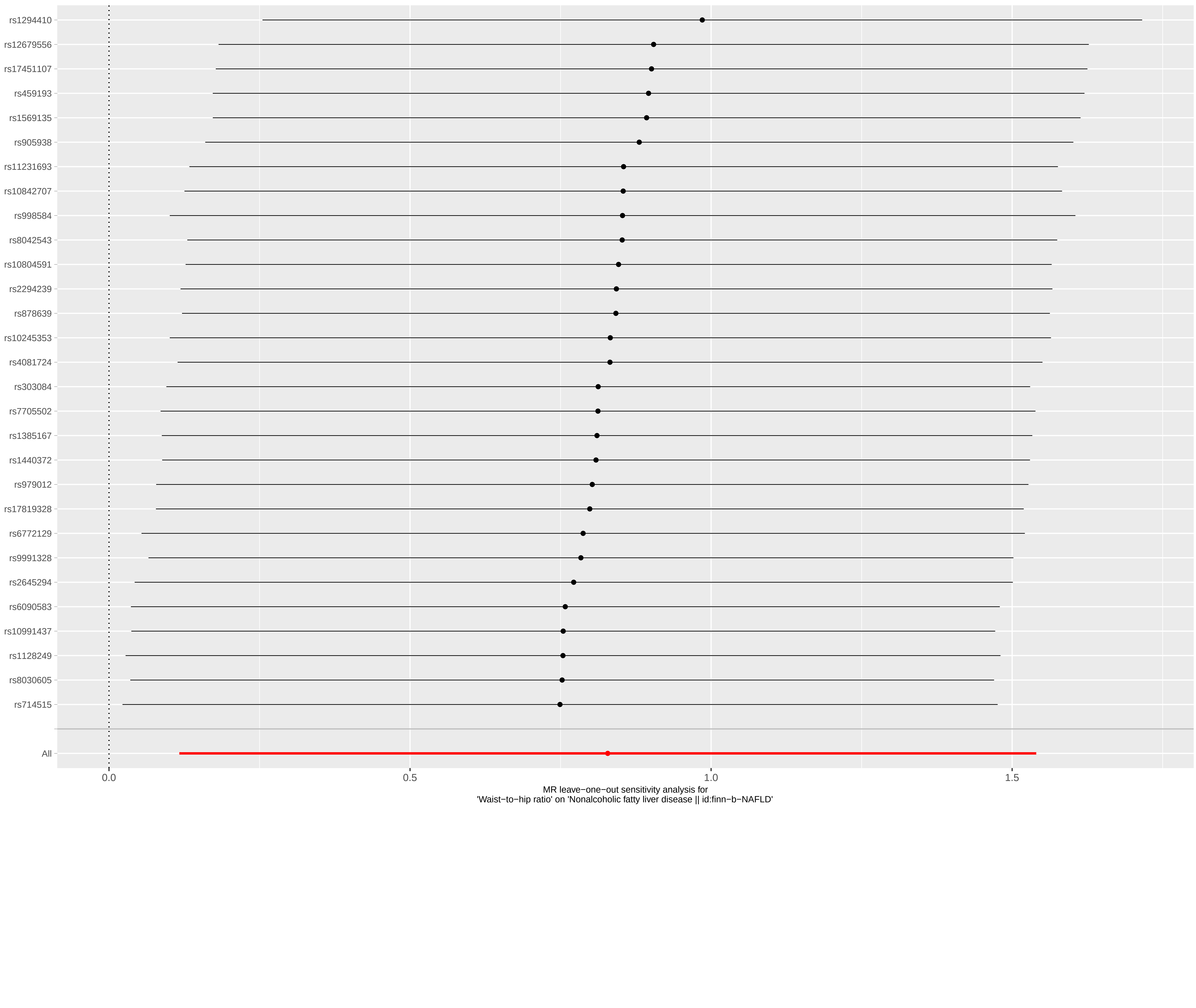

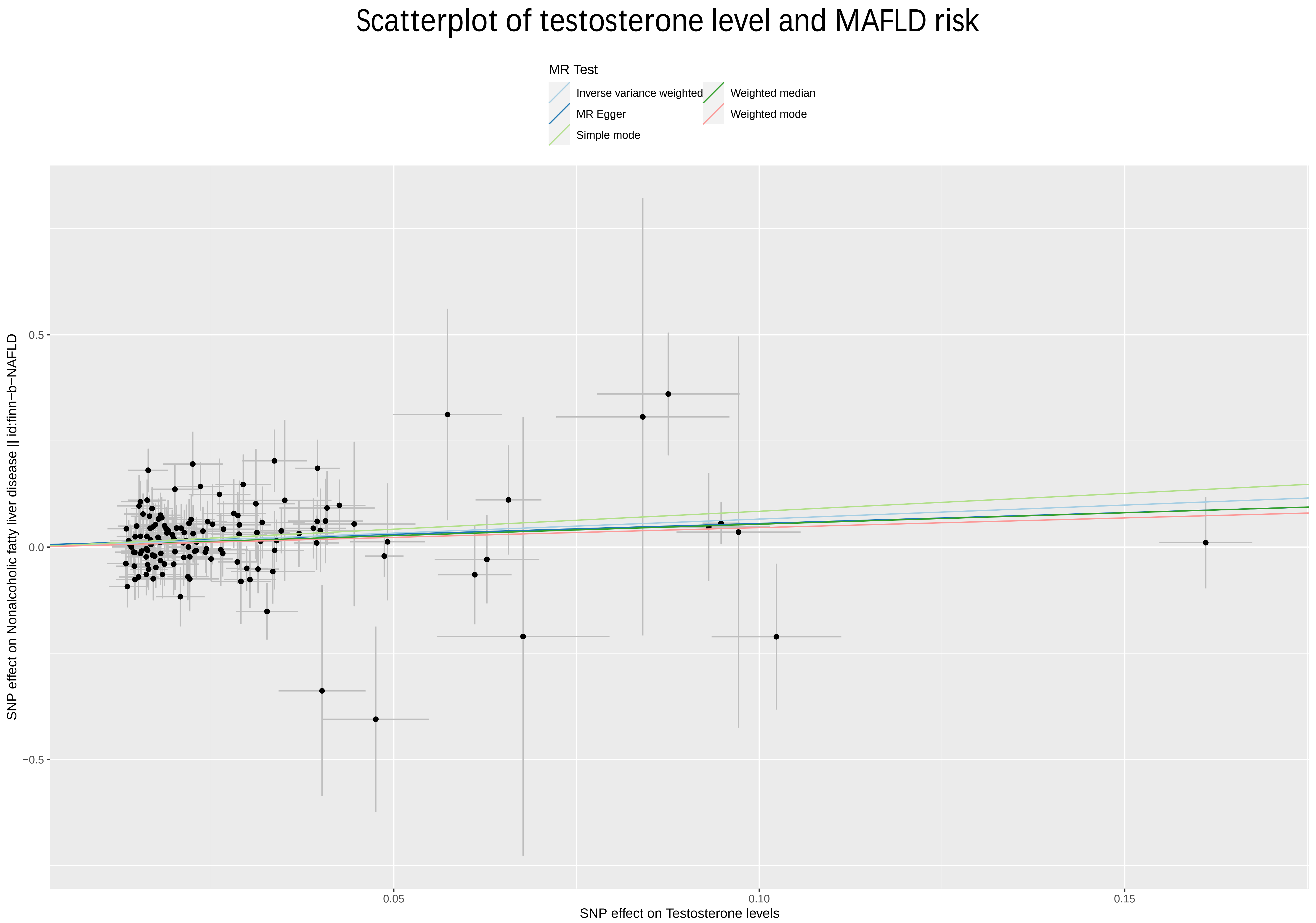
Scatterplot of testosterone level and MAFLD risk
MR Test
Inverse variance weighted
MR Egger
Simple mode
Weighted median
Weighted mode
SNP effect on Nonalcoholic fatty liver disease || id:finn−b−NAFLD
0.5
0.0
−0.5
0.05
0.10
0.15
SNP effect on Testosterone levels

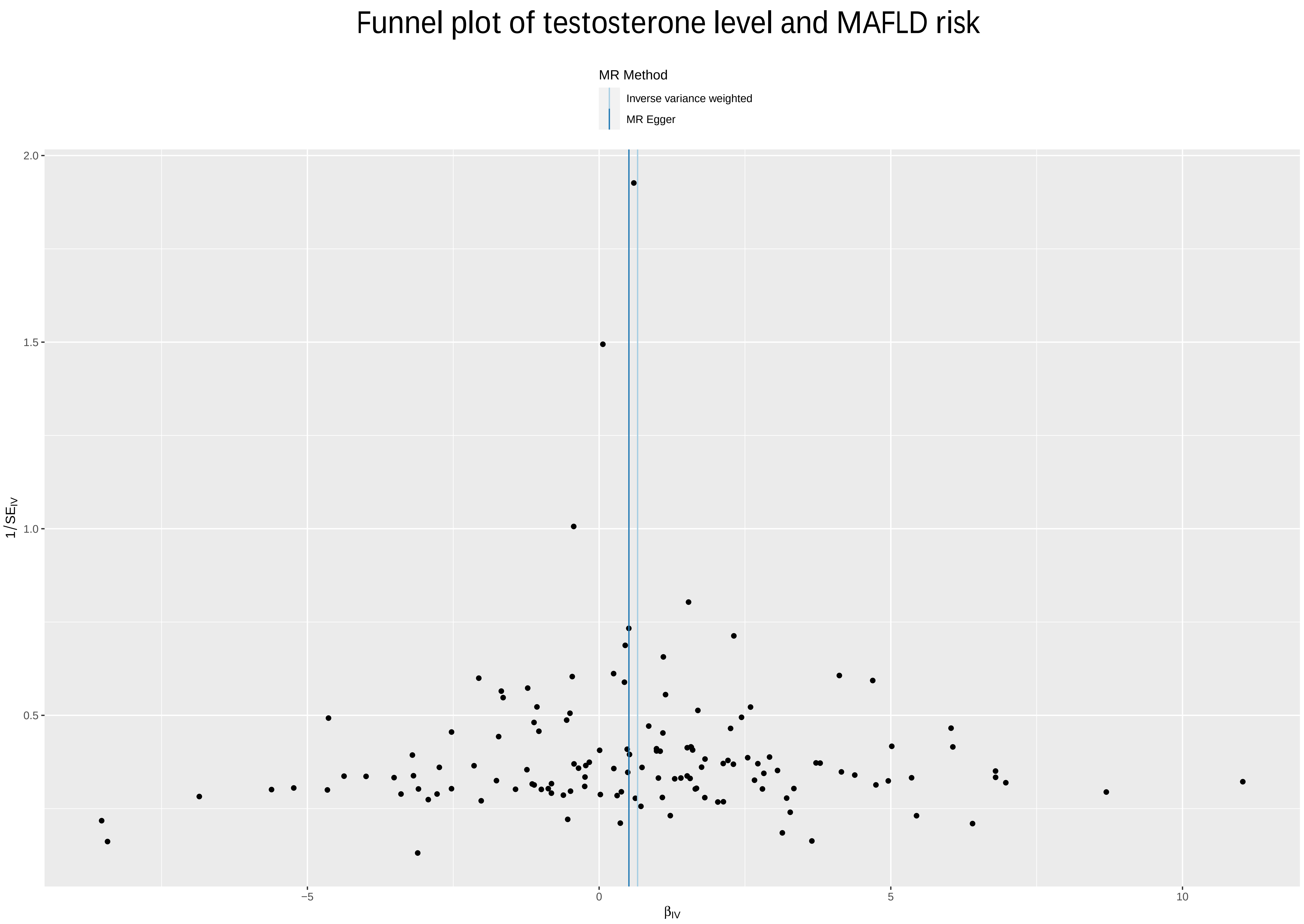

Funnel plot of testosterone level and MAFLD risk
MR Method
Inverse variance weighted
MR Egger
2.0
1.5
1.0
0.5
1/SE_IV
−5
0
5
10
β_IV

Forest plot of testosterone level and MAFLD risk

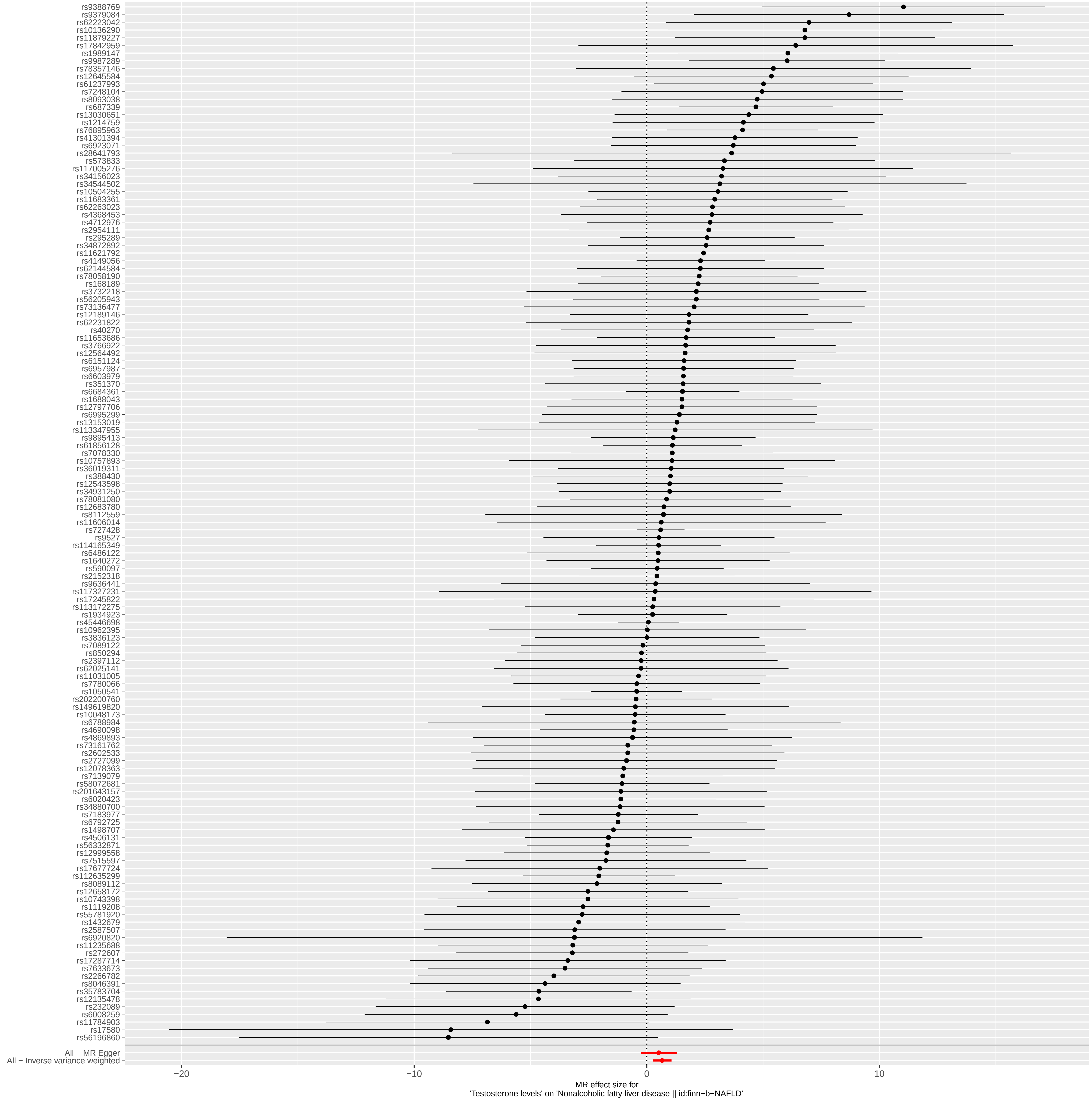

Sensitivity analysis of the "leave-one-out" method for testosterone level and MAFLD risk

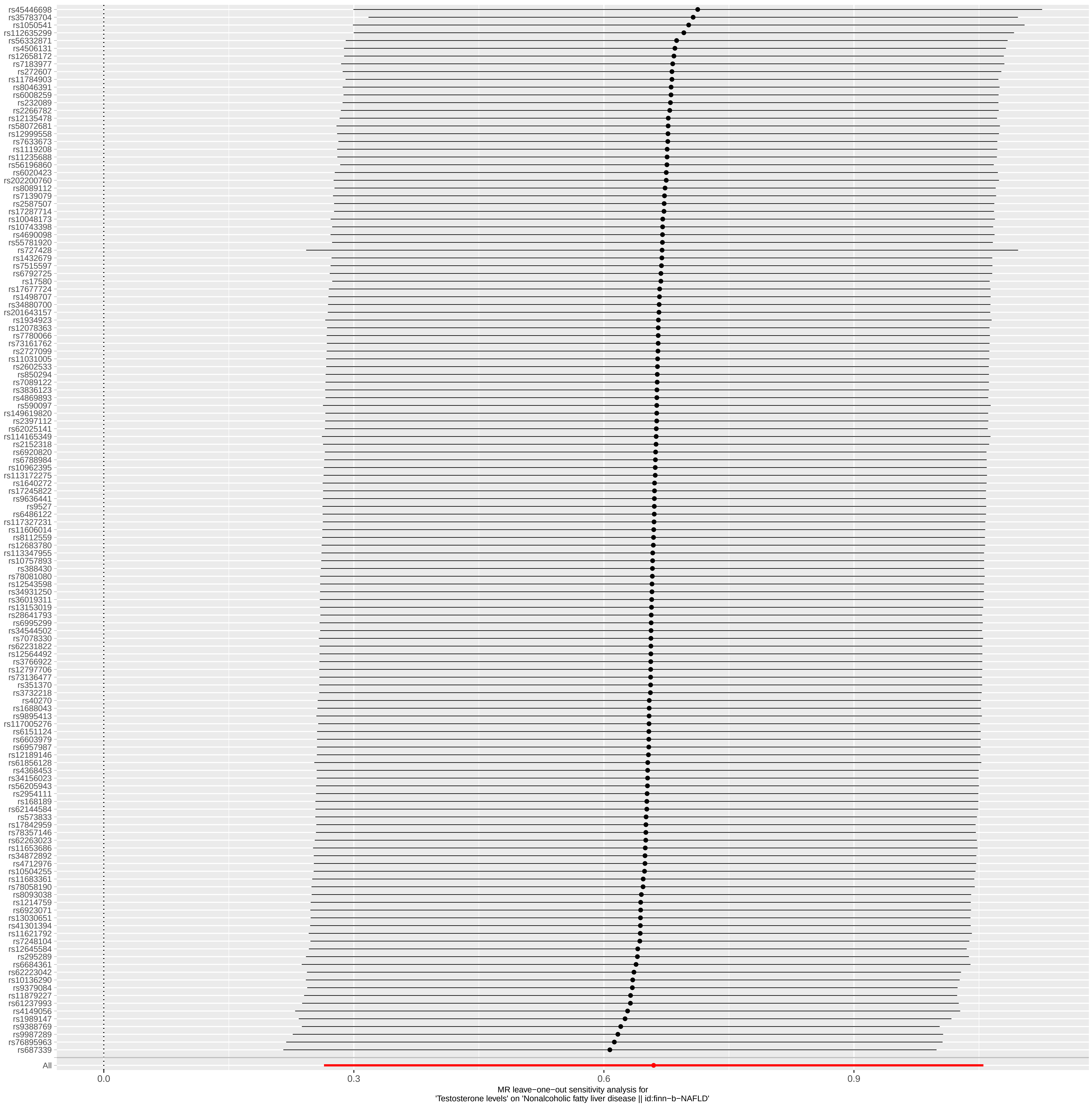

Supplement: Supplementary file 1 — MR results visualization charts [file 12889_2023_15467_MOESM1_ESM.pdf]
